# A bibliometric analysis of the global research on sofosbuvir

**DOI:** 10.12688/f1000research.12314.1

**Published:** 2017-08-22

**Authors:** Akram Hernández-Vásquez, Diego Rosselli

**Affiliations:** 1 Universidad Privada del Norte, Lima, 15434, Peru; 2 Department of Clinical Epidemiology and Biostatistics, Faculty of Medicine, Pontificia Universidad Javeriana, Bogotá, 11001000, Colombia

**Keywords:** Sofosbuvir, Hepatitis C, HCV, Bibliometric, Scientometric, Scientific production, Bibliographic Database, Network Analysis

## Abstract

In this article, we examine the research on sofosbuvir with a bibliometric analysis of global research production. The study of sofosbuvir has been a field of intense research in the past few years, with Latin American contributions playing a modest role. With continued drug development and approval of hepatitis C antivirals, research is expected to increase. Our findings will assist scholars and policy makers in their efforts to improve scientific research policies, with the goal of maximizing the access to treatments, especially in low and middle-income countries.

## Introduction

Hepatitis C virus (HCV) has a major impact on public health, with around 170 million people in the world being affected ^1^. In Latin America, the prevalence of hepatitis C has been estimated to be at around 1.6% of the adult population, and the most common genotypes are 1 and 3 ^2, 3^. The new treatments for HCV include direct-acting antivirals (DAAs), which shorten length of therapy, improve sustained virologic response rates, and minimize side effects ^4^.

Sofosbuvir is one of the most important DAAs in the market today, but high prices have led to a large increase in spending by health systems and can be a barrier to rapid global treatment, especially in low and middle-income countries ^5^. The identification of global research on DAAs might lead to new insights into the treatments of HCV and suggest research directions.

Based on the above, our study aimed to identify and explore the worldwide development of sofosbuvir research.

## Methods

We performed a bibliometric analysis using the original articles indexed in Web of Science. The search strategy used the following MeSH and non-MeSH terms in the title field: Sofosbuvir, Sovaldi, PSI 7977, and GS 7977. The validity of the search strategy was tested by manually reviewing retrieved articles. Bibliometric indicators were investigated by analyzing annual research output, languages, countries, journals, authors, institutions, and citations. Indicators were analyzed with the option “Analyze Results” and “Create Citation Report” in Web of Science. Author co-citation analysis (ACA) was presented as network visualization map using VOSviewer (version 1.6.4) techniques ^6^.

## Results

A total of 341 publications for the period of 2010–2017 (up to March 31, 2017) were retrieved and assessed. There were a total of 126 journals that published research on sofosbuvir. Twenty-four articles were economics-based. The three most prolific journals were Hepatology (31 articles), Gastroenterology (17), and Journal of Hepatology (17), responsible for 19.1% of the total publication output. The retrieved documents were published by 46 different countries. The largest contributors in absolute number of articles were USA (220), France (47), and Germany (43). Only one article was from Latin America (Brazil), and it was about sofosbuvir and Zika ^7^. The total number of authors for all articles was 2044. John G. McHutchison from Gilead Science (GS) published the most documents in this field (55), followed by William T. Symonds from GS (30), Diana M. Brainard from GS, and Eric Lawitz from Texas Liver Institute/University of Texas Health Science Center (28). Gilead Sciences (131), Bristol Myers Squibb (38), and Merck (27) were the three major funders. A total of 86 articles were from GS, 30 from Inova Fairfax Hospital, and 26 were from University of Texas. The sum of the citations related to the published articles was 10036. Average citations per item were 29.4, with an h-index of 46.

**Figure 1.**
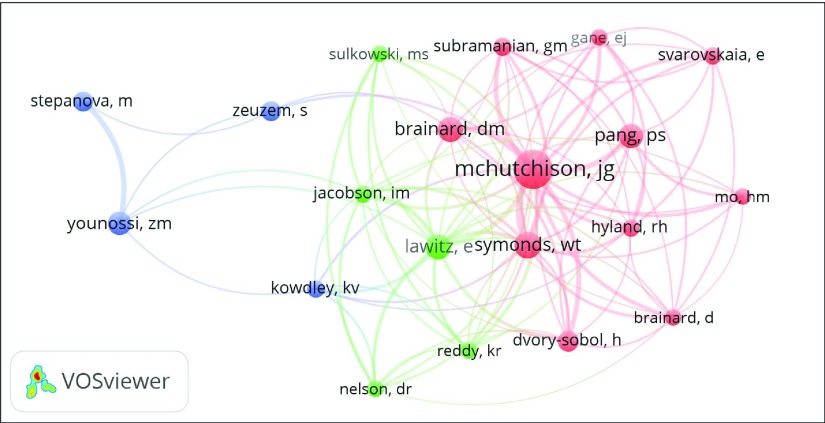
Author co-citation analysis with VOSviewer for sofosbuvir publications. Minimum of fifteen articles per author, 20 authors were included.

The number of authors included in the ACA was based on a minimum number of fifteen articles per author. The map produced included 20 authors distributed into three clusters (red, blue, and green), as shown in Figure 1. The red cluster included eleven authors (headed by John G. McHutchison from GS), the blue cluster included five (headed by Eric Lawitz from Texas Liver Institute/University of Texas Health Science Center), and the green cluster included four authors (headed by Zoibar Younossi from Inova Fairfax Hospital).

Data obtained from Web of Science CSV contained list of studies included. Click here for additional data file. Copyright: © 2017 Hernández-Vásquez A and Rosselli D 2017 Data associated with the article are available under the terms of the Creative Commons Zero "No rights reserved" data waiver (CC0 1.0 Public domain dedication).

## Conclusions

According to our bibliometric analysis, the study of sofosbuvir has been a field of intense research in the past few years. Developed countries have had an enormous impact on the global research in the field. Recently, interest has focused on the use of sofosbuvir to treat Zika infection, and an important contribution to the body of sofosbuvir research has been supported by its manufacturer. With continued drug development and approval of hepatitis C antivirals, research is expected to continue increasing.

## Data availability

The data referenced by this article are under copyright with the following copyright statement: Copyright: © 2017 Hernández-Vásquez A and Rosselli D

Data associated with the article are available under the terms of the Creative Commons Zero "No rights reserved" data waiver (CC0 1.0 Public domain dedication). 

**Dataset 1: Data obtained from Web of Science.** CSV contained list of studies included. Doi, 10.5256/f1000research.12314.d174424 ^8^
