# Supplementary material for: A bibliometric analysis of the global research on sofosbuvir
Source: F1000Res. 2017 Aug 22;6:1536. [Version 1] doi: 10.12688/f1000research.12314.1 (PMC5721933; doi:10.12688/f1000research.12314.1)
Supplement: Peer review history

# Peer review: Referee response for version 1

**DOI:** 10.5256/f1000research.13329.r28638

**Published:** 2017-12-07

**Authors:** Thiago Moreno L. Souza

**Affiliations:** 1 Laboratory of Immunopharmacology, Oswaldo Cruz Institute, Oswaldo Cruz Foundation (Fiocruz), Rio de Janeiro, Brazil; 2 National Institute for Science and Technology on Innovation on Diseases of Neglected Populations (INCT/IDPN), Center for Technological Development in Health (CDTS), Fiocruz, Rio de Janeiro, RJ, Brazil

The manuscript from Hernández-Vásquez and Rosselli reviews the state of art on sofosbuvir and HCV. This analysis leads to the notion that the contribution of Latin American Institutions to the field is marginal, which is likely an anticipated outcome. The authors move out of the main field of interest of the manuscript, HCV, to Zika virus (ZIKV) to highlight a Latin American contribution to the improved comprehension of the pharmacology of sofosbuvir.

 As sofosbuvir became an antiviral of major interest, not only towards HCV and ZIKV, but also for Dengue virus (DENV) ( https://www.ncbi.nlm.nih.gov/pmc/articles/PMC5524696/) – my suggestion is to compare the studies on the pharmacology of this drug on HCV and Flavivirus members. The study on ZIKV and sofosbuvir cited in this manuscript ( https://www.nature.com/articles/srep40920) was preceded by a pre-print, which was the first contribution to open the field of sofosbuvir against flaviviruses ( https://www.biorxiv.org/content/early/2016/07/06/061671; https://www.the-scientist.com/?articles.view/articleNo/46585/title/Sofosbuvir-Shows-Anti-Zika-Activity-In-Vitro/) – around six months after the ZIKV outbreak. I presume that the proposed comparison will demonstrate the installed capacity of response to novel (re)emergent viruses in Latin America.

I have read this submission. I believe that I have an appropriate level of expertise to confirm that it is of an acceptable scientific standard, however I have significant reservations, as outlined above.

# Peer review: Referee response for version 1

**DOI:** 10.5256/f1000research.13329.r26177

**Published:** 2017-09-25

**Authors:** Samy A. Azer

**Affiliations:** 1 Curriculum Development Unit, College of Medicine, King Saud University, Riyadh, Saudi Arabia

I read with great interest the above titled article. However there are several issues/problems that need to be improved.

**Abstract**

 The whole abstract should be rewritten. We need first to know the rationales of the study, or why is this study needed? What is the research question? What is the aim of the study? Then under state the search engine used and key words used in the search, and briefly state how did you process findings. Then state the bibliometric parameters that you aimed to study. State key results that answered your research question and stated under methods (follow the same sequence). Then state 2-3 lines summarizing key lessons learnt from your findings or what we can conclude from your study findings or take home messages. Ask your-self, “did the study answer my research question?” If yes, in what way...

 What are the bibliometric parameters that you examined? State them under methods and include the findings for each one.

 There are several redundant words in the abstract such as “In this study”, “we examined the research on sofosbuvir with a bibliometric analysis..” but you did not mention, even under methods in the manuscript, which type of research did you include in your analysis. Did you include only human or animal studies as well? Did you include only basic research or clinical research and clinical trials or both? Did you only include randomized controlled studies only or also you did include observational studies, controlled studies, reviews, and meta-analysis? Which of which did you include?

**Introduction**

 Please note the following regarding “Second paragraph” under Introduction:

- What do you mean by “most important DAAs”? You may omit this
- We need to know why did you chose Sofosbuvir and not other drugs in this group
- What are the advantages of Sofosbuvir over other drugs in this group and add references.
- Not clear about the sentence “high prices have led to a large increase in spending by health systems and can be a barrier to global treatment…” how this is related to the bibliometric analysis you are aiming at? Focus on the study purpose.
- State the rationale of the study
- State the bibliometric parameters you aim to analyze.
- State clearly the research question
- Add appropriate references for each item
- The statement “based on the above….” Is meaningless and should be omitted.

 

**Methods:**

- State the study design. Add a reference
- State the date of searching the Web of Science, and who conducted such search. Did both researchers share this responsibility?
- It is not clear what do you mean by MeSH and non-MeSH? Did you use PubMed as well? Explain why?
- What do you mean by PSI 7977 and GS7977, if these are the codes for the drug before its marketing. State this and the references for this.
- What were the inclusion and exclusion criteria?
- Did you include only basic research or clinical research and clinical trials or both? Did you only include randomized controlled studies only or also you did include observational studies, controlled studies, reviews, and meta-analysis? Which of which did you include?
- Did you include editorials? Did you include letter to the Editor? Did you include animal studies? Did you include review studies and meta-analysis? All these details are needed.
- What were the bibliometric parameters that you analyzed?
- I cannot see any description of how did you analyse the data gathered. How did you reach to agreement to include or not to include an article?
- Did you calculate the degree of agreement between researchers?

 

**Results**

- This section needs a lot of work
- The results should mirror the subtitles under methods
- Provide results for each subitem in the bibliometric analysis you did.
- The assessment is superficial and more in depth analysis is needed.
- Use subtitles
- You may create 2-3 tables summarizing key findings/analysis.
- What does Figure 1 mean?

 

**Discussion**

- I cannot see a section for “the discussion”. You should add a discussion.
- Remind the research of your research question, briefly state your key findings
- Discuss the agreement and disagreement with other researchers.
- Discuss the meaning of your findings.
- Discuss the limitation of the study
- Discuss the significance of your findings
- Future directions in this area

 

**Conclusion**

- This should be rewritten in light of your findings.
- Your statement about bibliometric analysis is misleading. I cannot see any bibliometric analysis from what I have read.

 

**References**

- Only 8 references. Please read the literature in this area and improve this section

 

**Supplementary data**

 I would advice also submitting the raw data of the material collected in a table showing the author (first author and year), title of article, journal detail, type of article (research, review, meta-analysis, article, editorial etc), and name of university/country involved.

I have read this submission. I believe that I have an appropriate level of expertise to confirm that it is of an acceptable scientific standard, however I have significant reservations, as outlined above.

## References

[1] Hernández-VásquezARosselliD: A bibliometric analysis of the global research on sofosbuvir. *F1000Research*.2017;6: 10.12688/f1000research.12314.1 10.12688/f1000research.12314.1 29259762PMC5721933

# Peer review: Referee response for version 1

**DOI:** 10.5256/f1000research.13329.r25248

**Published:** 2017-09-04

**Authors:** Juliana Gonçalves Reis

**Affiliations:** 1 Postgraduate Program in Public Health and Environment, National School of Public Health Sergio Arouca (ENSP/Fiocruz), Niterói, Brazil

Methods

- Was it necessary to clean and standardize the data?
- Describe the option of the bibliographic database wos.
- Why was it used a high limit for the number of documents per author?

 

 Results

- I suggest a chart of the distribution number of documents per year.
- I suggest a network of articles keywords with the description of the clusters.

 

 Conclusions

- What are the main gaps?
- What are the possibilities for future research?

I have read this submission. I believe that I have an appropriate level of expertise to confirm that it is of an acceptable scientific standard.
